# TTDB: a comprehensive Transcriptome Turnover Database for exploring mRNA stability

**DOI:** 10.1093/nar/gkaf1093

**Published:** 2025-10-29

**Authors:** Hao Jiang, Zhicheng Xu, Tong Li, Ji Wang, Zhi Xie

**Affiliations:** State Key Laboratory of Ophthalmology, Zhongshan Ophthalmic Center, Sun Yat-sen University, Guangdong Provincial Key Laboratory of Ophthalmology and Visual Science, Guangzhou 510060, China; State Key Laboratory of Ophthalmology, Zhongshan Ophthalmic Center, Sun Yat-sen University, Guangdong Provincial Key Laboratory of Ophthalmology and Visual Science, Guangzhou 510060, China; State Key Laboratory of Ophthalmology, Zhongshan Ophthalmic Center, Sun Yat-sen University, Guangdong Provincial Key Laboratory of Ophthalmology and Visual Science, Guangzhou 510060, China; State Key Laboratory of Ophthalmology, Zhongshan Ophthalmic Center, Sun Yat-sen University, Guangdong Provincial Key Laboratory of Ophthalmology and Visual Science, Guangzhou 510060, China; Department of Pathology, University of Cambridge, Cambridge CB2 1QP, United Kingdom; State Key Laboratory of Ophthalmology, Zhongshan Ophthalmic Center, Sun Yat-sen University, Guangdong Provincial Key Laboratory of Ophthalmology and Visual Science, Guangzhou 510060, China

## Abstract

The regulation of messenger mRNA turnover is a critical cellular process that dictates gene expression levels. High-throughput sequencing technologies have enabled the measurement of mRNA decay rates on a transcriptome-wide scale. However, this vast amount of data is dispersed across numerous publications, and it is cumbersome to harmonize and compare. To address this, we have developed the Transcriptome Turnover Database (TTDB), a centralized and comprehensive resource for genome-wide mRNA stability data. TTDB integrates 198 high-throughput datasets from 57 publications, with an average 12 544 genes per dataset, across five species: human, mouse, zebrafish, fruit fly, and yeast. The database not only provides experimentally determined and computationally standardized mRNA half-lives and decay rates, but also offers precomputed annotations for each transcript, including GC content, minimum free energy of mRNA secondary structure, and counts of regulatory elements like upstream AUGs and AU-rich elements. The user-friendly web interface allows users to intuitively browse, search, visualize, and download data by study, sample, or gene. TTDB is publicly accessible at https://sysbio.gzzoc.com/ttdb/index.html.

## Graphical Abstract

**Graphical Abstract:**
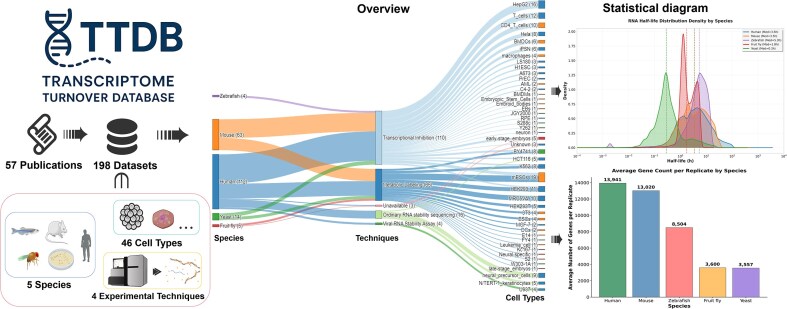

## Introduction

The steady-state level of messenger RNA (mRNA) is determined by the balance between its synthesis (transcription) and degradation (turnover). The rate of mRNA turnover is a highly regulated process that allows cells to rapidly alter their proteome in response to developmental cues and environmental stimuli [1]. Dysregulation of mRNA stability is widely implicated in human cancer and diseases. For example, a common risk-associated genetic variant in age-related macular degeneration is known to destabilize the *ARMS2* mRNA, leading to disease susceptibility [2].

In recent years, the development of high-throughput methods, such as metabolic labeling with 4-thiouridine (4sU-seq) and transcriptional inhibition, has enabled the measurement of mRNA decay rates for thousands of genes simultaneously [3, 4]. This has resulted in a wealth of data scattered across the scientific literature. However, utilizing these data effectively is challenging due to variations in experiments, data processing, and reporting formats. A researcher who seeks and compares the stability of genes of interest across different cell types or conditions must undertake the arduous task of finding, downloading, reprocessing, and reanalyzing multiple datasets.

While a few resources related to RNA stability exist, they are typically specialized. For instance, some databases focus on specific RNA types, such as long non-coding RNAs in mouse [5], or on mRNA half-lives within a single context, like human T-cells [6]. Other platforms serve different functions; tools such as mRNAStab [7] are designed to analyze user-supplied data rather than acting as integrated data repositories, while databases like RNApathwaysDB [8] qualitatively describe the enzymes and components of decay mechanisms instead of providing large-scale quantitative experimental data. To our knowledge, there is no database to systematically collect, standardize, and integrate this quantitative data across multiple key model organisms, addressing the critical need for a platform to facilitate cross-species and cross-conditional comparative studies.

**Figure 1.**
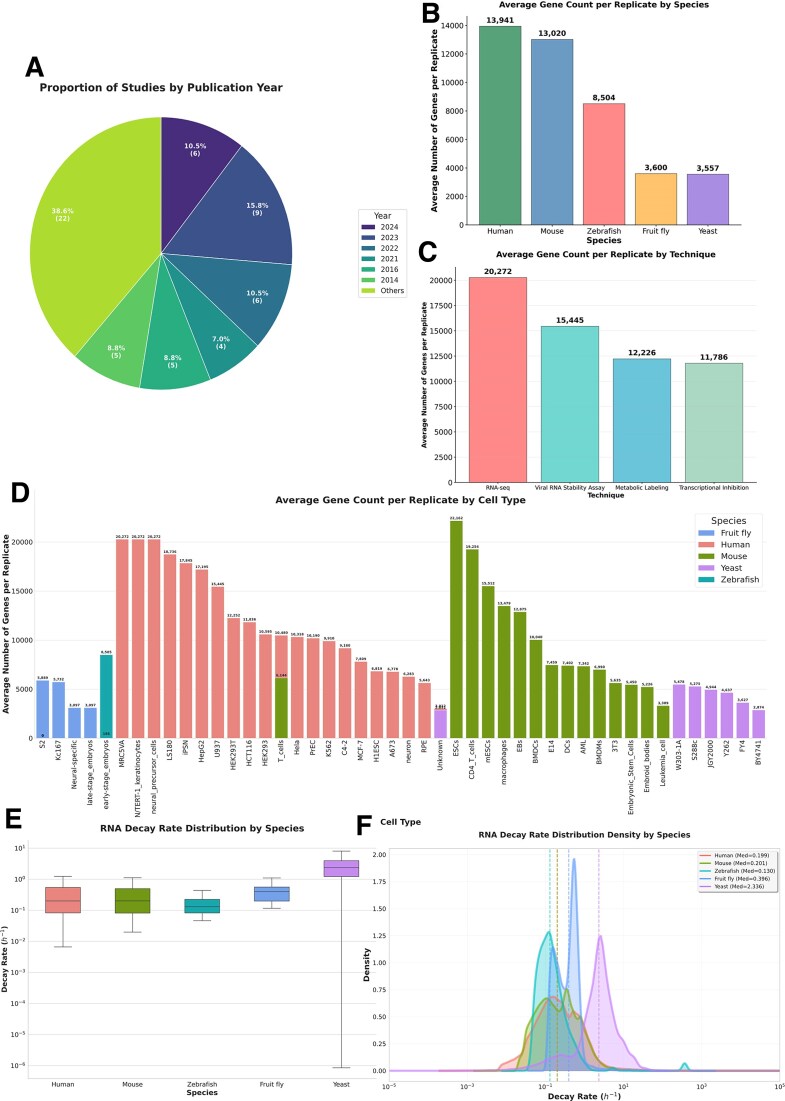
Statistical overview of the TTDB database. A comprehensive summary of the data curated in the TTDB. (**A**) Pie chart showing the proportion of the 57 curated studies, categorized by their publication year. (**B**) Bar chart displaying the average number of genes quantified per replicate, categorized by species. (**C**) Bar chart showing the average number of genes quantified per replicate, categorized by the experimental technique used to measure mRNA stability. (**D**) Vertical bar chart comparing the average number of genes quantified per replicate across various cell types, which are grouped and color-coded by species. (**E**) Box plots showing the distribution of mRNA decay rates (in units of h^−1^) for each of the five species included in TTDB. (**F**) Density plots providing an alternative visualization of the mRNA decay rate distributions for each species. The median decay rate for each species is indicated by a dashed vertical line.

**Figure 2.**
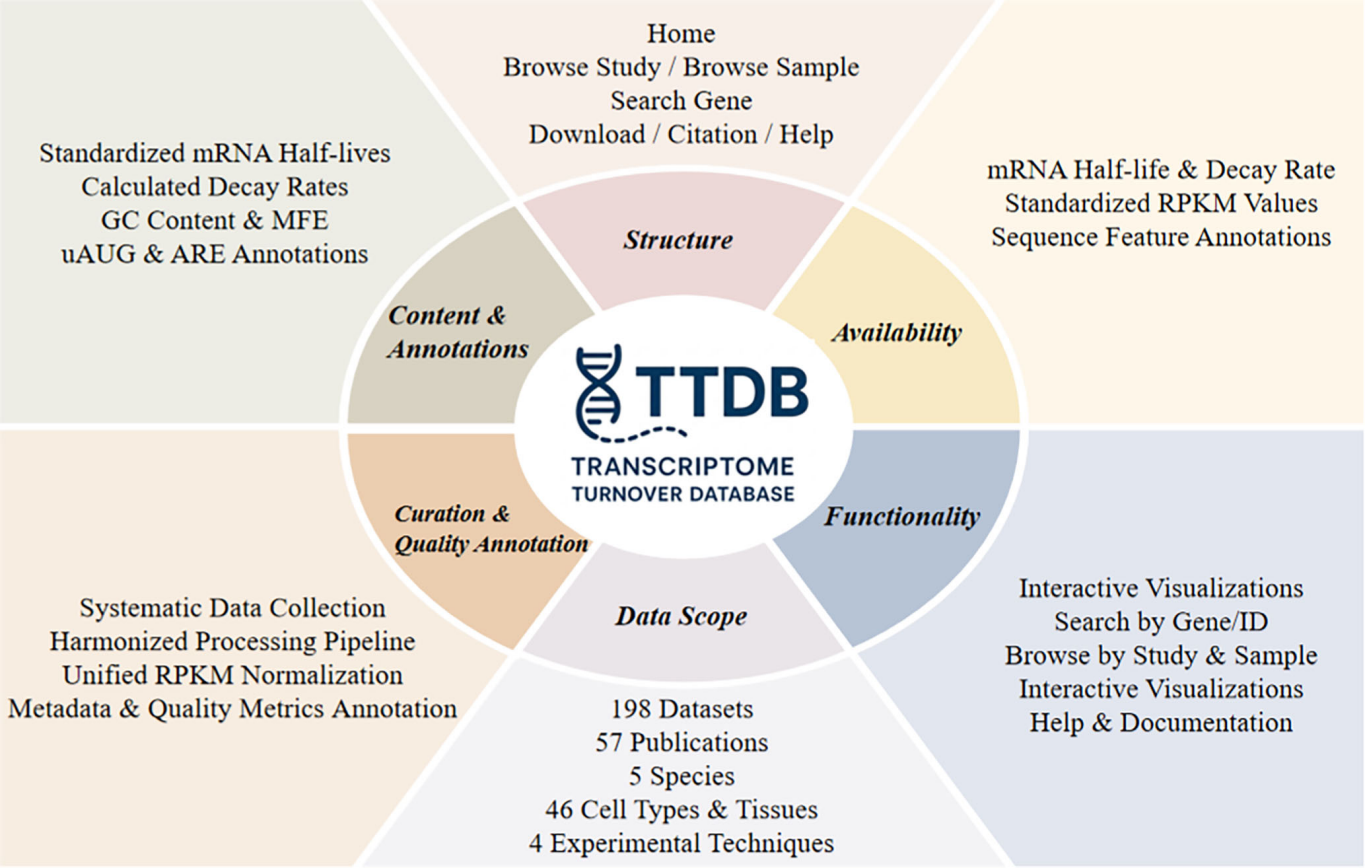
Overview of TTDB.

To address this gap, we developed the Transcriptome Turnover Database (TTDB), aiming to serve as a comprehensive platform that consolidates information from all the high-throughput mRNA stability studies available in the public domain to date, and provides a suite of tools for querying and exploring the integrative information. Figure 1 provides a statistical overview of the data curated in TTDB, while Fig. 2 provides a schematic overview of its data and contents. TTDB aims to facilitate comparative analysis of gene stability across diverse studies, samples, and biological conditions and to support hypothesis generation for a broad community of biologists and bioinformaticians.

## Data sources

The studies included in TTDB were identified through a systematic search and curation strategy. We performed advanced searches in the PubMed and Gene Expression Omnibus (GEO) databases for the five target species (human, mouse, zebrafish, fruit fly, and yeast) using a comprehensive query combining keywords for the biological process and experimental scale, such as (“mRNA stability” OR “RNA decay” OR “mRNA half-life”) and (“high-throughput” OR “transcriptome-wide” OR “RNA-seq”). The search results were then manually curated. A study was included only whether it was explicitly described as a transcriptome-wide measurement of mRNA stability using an established high-throughput methodology and provided either the final processed half-life/decay rate data or the complete time-course sequencing data (e.g. via a GSE accession number) required for recalculation.

Following this procedure, data in TTDB was manually curated from 57 published studies. A complete list of the included studies is provided in Supplementary Table S1. The current version includes 198 distinct high-throughput datasets covering 46 different cell types and tissues across human, mouse, zebrafish, fruit fly, and yeast, where the average genes per database in each species were 13 942, 13 020, 8504, 3600, and 3557, respectively.

## Data processing and annotation

**Figure 3.**
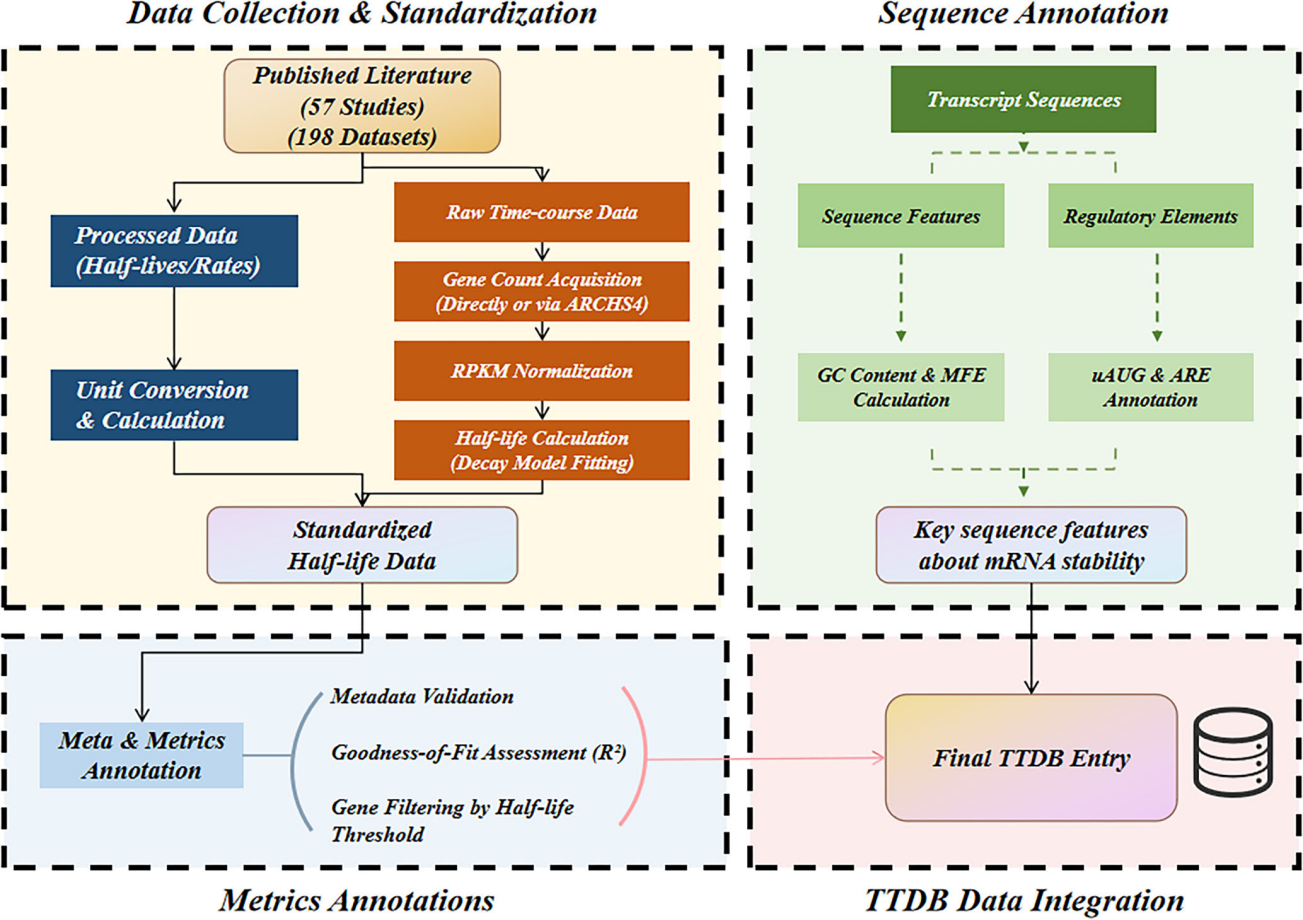
The data processing and curation workflow for TTDB.

The data are processed and annotated in three levels (Fig. 3).

### Data standardization

Data from heterogeneous sources were standardized, ensuring comparability across all datasets. For studies that directly reported processed half-life values or decay rates, we performed unit conversion (e.g. minutes to hours) and, where necessary, calculated half-lives (*t*_1/2_) from decay constants (*k*) using the formula:


\begin{eqnarray*}
{{t}_{_{1/2}}} = \frac{{1{\mathrm{n}}(2)}}{k}.
\end{eqnarray*}

For datasets where only time-course sequencing data was available, we established a unified processing pipeline. If raw gene-level counts were provided in the publication, they were used directly. Otherwise, for studies providing only a GEO Series (GSE) accession number, we obtained the corresponding gene-level counts for each time-point sample (GSM) from the ARCHS4 resource [9]. All time-course count data was subsequently normalized to Reads Per Kilobase of transcript per Million mapped reads (RPKM). Other normalization metrics, such as Transcripts Per Million, were not used because a substantial portion of the historical datasets integrated into TTDB only provided precalculated RPKM values, with their raw sequencing data being inaccessible.

To ensure this method is rigorous and reproducible, the “principal transcript” was systematically identified based on the widely accepted standard defined by Ensembl (Release 115, September 2025) [10] and the GENCODE consortium [11]. We adopted a clear hierarchy of evidence: priority was given to isoforms annotated by the Matched Annotation from NCBI and EBI (MANE) project, followed by the APPRIS principal isoform annotations, and finally the transcript support level. This approach ensures a single, biologically representative isoform is used for each gene.

Finally, the resulting standardized time-series expression data for each gene was fit to a first-order exponential decay model to calculate its half-life.

### Metrics annotations

To enrich the primary stability data, each entry in TTDB is annotated with several key metrics that provide experimental context, data quality assessment, and benchmarks for interpretation. These annotations include:

1. Metadata: Each entry is marked with its species and cell type by cross-referencing authoritative databases to provide essential experimental context.
2. Goodness of fit: For datasets derived from time-course experiments, we calculated the coefficient of determination (*R*^2^) to evaluate how well a transcript’s decay fits the first-order exponential model. These *R*^2^ values serve as indicators for users to independently perform quality-based assessments.
3. Stability thresholds: To help users classify transcripts, we provide a descriptive threshold for each species at the 75th percentile (upper quartile) of all half-lives in TTDB [12]. Within the database interface, genes with half-lives exceeding this value are explicitly annotated with this threshold number for quick identification.

It is important to note that this threshold is intended as a descriptive guide for user convenience, following the practice of prior bioinformatics resources, rather than a strict biological cutoff. This allows for streamlined functional comparisons between inherently stable and more transient transcripts. We emphasize that users can easily define their own custom stability thresholds for more specific analyses using the complete downloadable datasets.

### Sequence annotation

To facilitate deeper analysis, we annotated the main transcript of every gene with several sequence features known to influence mRNA stability.

1. GC content and minimum free energy (MFE): The sequence for each transcript was used to calculate its GC content and predict its secondary structure MFE. GC content for each transcript was calculated from its sequence using a custom Python script. The MFE of the predicted secondary structure was subsequently calculated using the RNAfold program from the ViennaRNA Package 2.0 [13] with default energy parameters.
2. Regulatory elements: We annotated two key classes of regulatory elements. First, we identified and counted upstream AUG (uAUG) codons within the 5′ UTR of each principal transcript. This was achieved using a custom Python script that implemented a direct pattern-matching algorithm to scan for all “ATG” start codons, a fundamental approach in uORF annotation [14]. We chose this method to ensure a transparent and precise count of canonical uAUGs, as predicting uORF functionality was beyond the scope of our annotation. The presence of uAUGs is a well-established feature of translational control, often acting to repress the synthesis of the primary protein [15]. Second, AU-rich elements (AREs) were annotated at the gene level by leveraging the comprehensive AREsite2 database [16]. This approach was chosen to match the gene-level resolution of our stability data. AREsite2 allows for the detailed investigation of AU-, GU-, and U-rich motifs across the entire transcriptome, including 5′ UTRs, coding sequences, and introns, rather than being restricted to the 3′ UTR. We queried the database for genes containing canonical ARE motifs (e.g. the “ATTTA” pentamer and its variants), which are critical *cis*-acting signals that recruit various RNA-binding proteins to modulate mRNA stability [17].

## Implementation

TTDB is built on a standard LAMP (Linux, Apache, MySQL, PHP) architecture. The backend data are managed by a MySQL database, with server-side scripting handled by PHP to process user queries and retrieve data. The web interface is constructed with standard HTML5, CSS3, and JavaScript, utilizing the Bootstrap framework for responsive design and styling. Client-side interactivity is enhanced through the jQuery library and dynamic data tables are rendered using the Bootstrap Table plugin. Interactive data visualizations, such as charts and plots, are powered by the Plotly.js and D3.js libraries. The entire system is hosted on an Apache web server.

## Database content

The TTDB web interface is designed for efficient data exploration through “Browse,” “Search,” and “Download” pages.

### Search gene

The “Search Gene” page is the entry point for investigating a specific gene. Users can enter a gene symbol or Ensembl ID. The results page displays half-life measurements for that gene across different studies and samples, along with the associated cell type, experimental technique, experimental conditions, and precomputed sequence annotations.

### Browse by study or by sample

To explore the data from a broader perspective, users can use the “Browse study” and “Browse sample” pages. These interfaces allow users to filter the entire database by study, keywords, species, cell type, or experimental techniques, providing a comprehensive overview of the available data and allowing for easy navigation to specific datasets of interest.

### Data download

All the data in TTDB, including the curated stability measurements and sequence annotations, is available for bulk download from the “Download” page. Data is provided in both comma-separated value and JavaScript object notation formats to accommodate a wide range of user workflows. The downloadable files contain comprehensive information for each measurement, including gene identifiers (Symbol and Ensembl ID), species, half-life and decay rate values, experimental context (study ID, sample name, and cell type), and precomputed annotations (e.g. GC content, MFE, and uAUG count). This detailed format allows for custom bioinformatic analyses, such as comparing stability between specific gene sets or conditions, and seamless integration with local datasets. To enhance clarity and usability, the header of each downloadable file includes a detailed description of each data field.

To facilitate deeper analysis and validation, direct links to the original publication (via PMID) and, where available, the raw sequencing data (e.g. via GEO series accession number) are provided for each study.

## Use case

**Figure 4.**
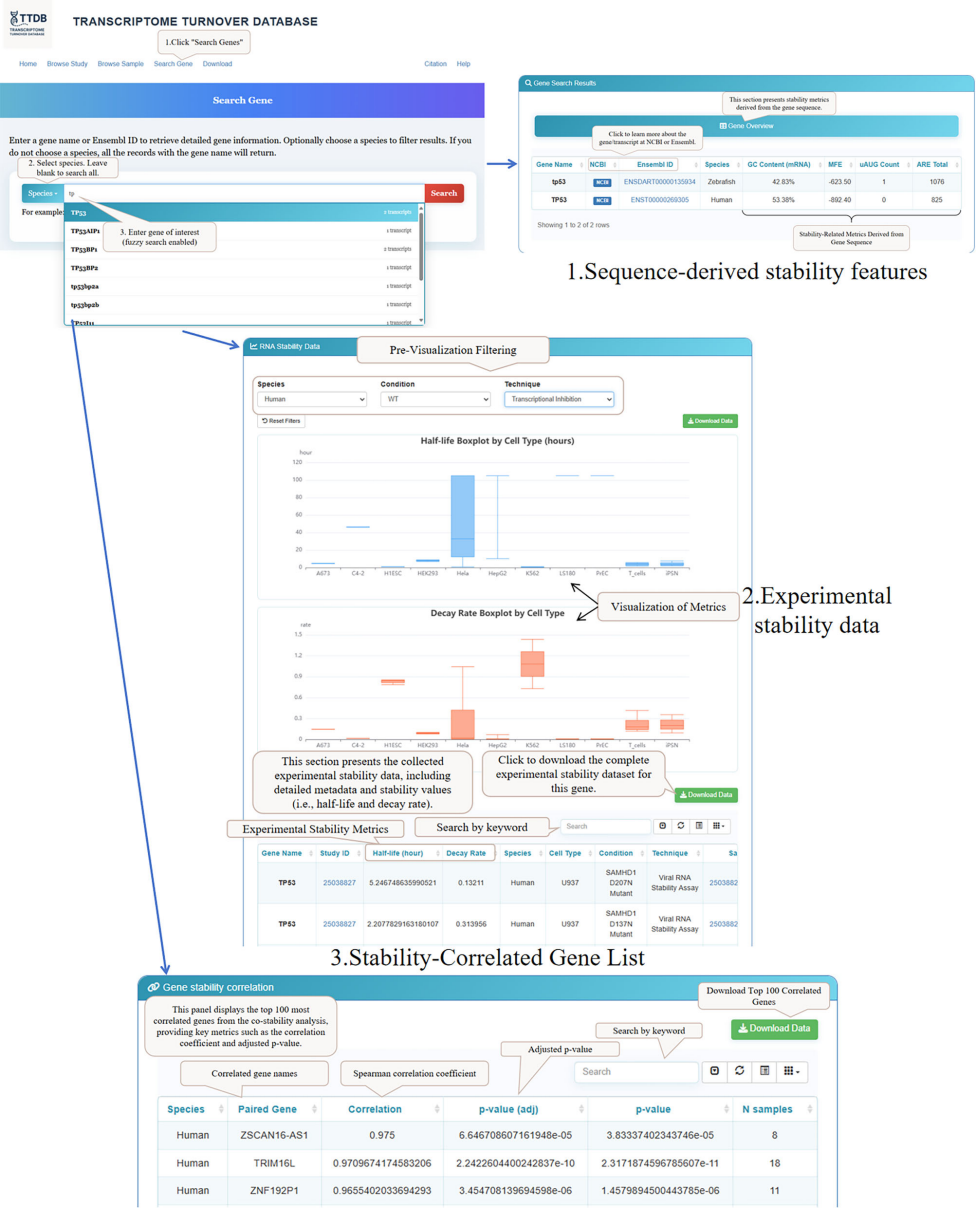
Use case demonstrating a typical user journey for the gene TP53 in TTDB. The workflow illustrates how a user can retrieve and analyze multi-layered data for a gene of interest. (1) Sequence-derived stability features: After searching for a gene, the top panel summarizes precomputed sequence annotations known to influence mRNA stability, such as GC content, MFE, and counts of uAUGs and AREs. (2) Experimental stability data: The central panel provides all curated experimental measurements. It includes an interactive boxplot for visualizing the distribution of half-lives across different conditions and a detailed data table aggregating stability values from various studies. (3) Stability-Correlated Gene List: The bottom panel displays the top 100 genes with the most correlated mRNA stability to the queried gene, providing Spearman correlation coefficients and adjusted *P*-values to facilitate the exploration of co-regulatory networks.

To illustrate a typical application of TTDB, we demonstrate an end-to-end user journey for investigating the human tumor suppressor gene TP53 (Fig. 4). Upon entering “TP53” into the “Search Gene” tool, the user is directed to a comprehensive results page that integrates multiple layers of information.

First, a summary panel presents key pre-computed, sequence-derived stability features (Fig. 4, panel 1). This allows for a quick assessment of sequence characteristics known to influence mRNA stability, such as GC content, MFE of the predicted secondary structure, and counts of regulatory elements like uAUGs and AREs.

Second, the user can explore the curated experimental stability data for TP53 (Fig. 4, panel 2). This section features an interactive boxplot that provides a visual summary of the half-life and decay rate distributions across different experimental conditions and techniques. Below the visualization, a detailed data table aggregates all available stability measurements for

TP53 from multiple independent studies, allowing for rapid comparison of its turnover rates across various cell types (e.g. U937 versus HepG2) and under different genetic perturbations (e.g. WT versus SAMHD1 mutant).

Finally, to explore potential coregulatory relationships, the page includes a “Stability-Correlated Gene List” (Fig. 4, panel 3). This panel interactively displays the top 100 genes whose mRNA stability is most correlated with that of

TP53. The table provides the Spearman correlation coefficient, the adjusted *P*-value, and the number of overlapping samples for each gene pair, facilitating hypothesis generation about coregulated mRNA decay pathways. This integrated view, from intrinsic sequence features to experimental data and co-stability networks, highlights the value of TTDB in accelerating biological discovery.

## Discussion

The regulation of mRNA stability is a cornerstone of gene expression, yet system-level insights have been hindered by the fragmentation of high-throughput data. TTDB directly addresses this critical barrier by creating the first systematically standardized resource for cross-species comparative analysis of mRNA decay. A key advance of TTDB is its function as a hypothesis-generation engine. By enriching decay rates with pre-computed annotations of *cis*-regulatory elements like uAUGs and AREs, the database allows researchers to rapidly connect stability patterns with potential underlying mechanisms. Furthermore, the inclusion of precomputed gene co-stability correlations allows users to explore potential coregulatory relationships, moving beyond the analysis of individual genes and simple data aggregation.

TTDB will be continuously updated as more datasets become publicly available. We have a clear roadmap for future development. Near-term goals include implementing in-browser statistical comparison tools to allow users to directly compare half-lives between selected conditions, and adding more direct links to external resources such as AREsite2 and the source GEO Series entries to improve data connectivity. Mid-term goals include incorporating isoform-resolved half-life data as it becomes more widely available, which will capture a deeper layer of posttranscriptional regulation. We also plan to integrate additional regulatory layers, such as data on RNA-binding protein binding sites, miRNA targets, and m^6^A modifications, to create a more comprehensive resource for studying the grammar of mRNA stability. By removing technical barriers and providing a unified exploratory platform, TTDB empowers the scientific community to investigate the complex grammar of posttranscriptional regulation and accelerate discovery.

## Supplementary Material

gkaf1093_Supplemental_File

## Data Availability

TTDB is freely accessible to all users without any login requirements at https://sysbio.gzzoc.com/ttdb/index.html.
